# Imputed HLA typing as a practical approach to molecular mismatch risk stratification in kidney transplantation

**DOI:** 10.3389/ti.2026.16964

**Published:** 2026-07-23

**Authors:** Alice Si Hua Ho, A. Vathsala, Hersharan Kaur Sran, Matthew Ross D’Costa, Zi Yun Chang, Ada Pei Yu Ng, Amy Lim, Wee-Kun Koh, Emmett Tsz Yeung Wong

**Affiliations:** 1 Department of Medicine, Yong Loo Lin School of Medicine, National University of Singapore, Singapore, Singapore; 2 Faculty of Medicine, School of Public Health, Imperial College London, London, United Kingdom; 3 National University Centre for Organ Transplantation, National University Hospital, Singapore, Singapore

**Keywords:** human leukocyte antigen, imputation, kidney transplantation, molecular mismatch, next-generation sequencing

Dear Editors,

Human leukocyte antigen (HLA) compatibility between donor and recipient is a key determinant for successful transplant outcomes [1]. Compared to conventional whole-antigen mismatch, molecular mismatch (mMM) more accurately predicts the risk of donor-specific antibody (DSA) development and allograft rejection in kidney transplant recipients [2]. Specifically, HLA-DR/DQ single-molecule eplet mismatch has been validated across multiple cohorts as a prognostic biomarker for primary alloimmunity [3]. However, eplet analysis requires high-resolution genotyping, which is often unavailable in historical cohorts and under-resourced settings. Although imputed haplotypes have shown inaccuracies, particularly in non-Caucasian populations and class II alleles [4, 5], imputation may preserve clinically meaningful mMM risk classification [6].

We investigated the impact of imputation on mMM risk assessment in an ethnically diverse cohort of Southeast Asian kidney transplant recipients to demonstrate proof of concept for a cost-effective alternative to high-resolution HLA typing, potentially applicable in resource-limited settings.

This single-center cohort comprised 32 living- and 19 deceased-donor adult kidney transplant pairs transplanted between September 2023 and November 2024, yielding 97 unique HLA samples, as five of the deceased donors each donated to two recipients. The cohort comprised predominantly Chinese (54%), followed by Malay (20%) and Indian (17%). Approval was obtained from the NHG Domain Specific Review Board (2024/00118), and the study complied with the Declaration of Helsinki.

Recipients and donors underwent high-resolution HLA genotyping by next-generation Sequencing (NGS; AllType, OneLambda, Canoga Park, CA). We transformed the NGS data, retaining the first field and removing all subsequent fields. Serological splits were considered for HLA-B*14, -B*15, -B*40, -B*55, -B*56, -C*03, -DRB1*03, and -DQB1*03. No individual had an HLA-DRB1*01:03 allele. This dataset was then imputed using HaploStats, which derives the most probable alleles based on haplotype frequencies in reference populations in the National Marrow Donor Program (NMDP) 2014 full dataset, to generate two-field genotypes for HLA-A, -B, -C, DRB1, -DRB345, and -DQB1 loci, selecting reference panels best aligned with each individual’s self-identified race. Alleles from the top-ranked phased genotype were selected across all HLA loci. The most probable HLA-DQA1 alleles were assigned using published haplotype frequency standards describing HLA-DRB1-DQB1-DQA1 associations [7, 8]. Null alleles associated with common haplotypes were considered, including the DRB1*07:01-DRB4*01:03N-DQB1*03:03 and DRB1*15:02-DRB5*01:08N-DRB5*01:02 haplotypes. HLA-DP was excluded from Haplostats because HLA-DP typing has historically been limited and inconsistent. HLA-DP also has higher recombination rates and weak linkage disequilibrium with HLA-DR and HLA-DQ, making phasing of HLA-DP from HLA-A-B-C-DR-DQ haplotypes unreliable.

Single-molecule eplet mismatch was evaluated at each locus using HLAMatchmaker (ABC version 4.0 and DRDQDP version 2.2), except HLA-DP. Recipients were categorized into three alloimmune risk groups according to thresholds previously published by Wiebe et al. [2]: Low-risk (maximum HLA-DR eplet mismatch <7 and HLA-DQ <9), Intermediate-risk (any HLA-DR and maximum HLA-DQ 9-14), and High-risk (any HLA-DR and maximum HLA-DQ ≥15). We quantified the concordance of alleles and mMM risk categories using weighted kappa coefficients, and the agreement of single-molecule eplet mismatches using Bland–Altman plots.

Allele-level concordance between NGS and imputed alleles was 80% (95% CI 74%–85%, Cohen’s κ = 0.77, 95% CI 0.71–0.84) for HLA-A, 89% (95% CI 83%–92%, κ = 0.88, 95% CI 0.82–0.93) for HLA-B, 92% (95% CI 87%–95%, κ = 0.91, 95% CI 0.87–0.95) for HLA-C, 83% (95% CI 77%–88%, κ = 0.82, 95% CI 0.76–0.87) for HLA-DRB1, 60% (95% CI 54%–67%, κ = 0.54, 95% CI 0.47–0.61) for HLA-DRB345, 86% (95% CI 80%–90%, κ = 0.84, 95% CI 0.78–0.89) for HLA-DQB1, and 88% (95% CI 83%–92%, κ = 0.87, 95% CI 0.82–0.92) for HLA-DQA1. Overall concordance was 87% (95% CI 84%–89%, κ = 0.87, 95% CI 0.84–0.89) and 79% (95% CI 76%–82%, κ = 0.79, 95% CI 0.76–0.82) for Class I and Class II alleles, respectively. The most frequently mis-imputed alleles were DRB4*01:03 (40/40, 100%) and DQB1*02:02 (12/12, 100%). Re-imputing DRB4*01:01 as DRB4*01:03 improved HLA-DRB345 concordance to 80%.

Despite allele-level inaccuracies, pairwise comparisons showed strong agreement between NGS- and imputation-derived single-molecule eplet mismatch counts at most loci (R^2^ > 0.95) except HLA-DRB345 (R^2^ = 0.67, Figure 1). HLA-DR/DQ mMM risk classification was generally consistent between NGS and imputed genotyping. By NGS, 12 (24%) donor-recipient pairs were classified as low risk, 25 (49%) as intermediate risk, and 14 (27%) as high risk. All recipients classified as low- or high-risk by NGS were similarly classified by imputation. Reclassification occurred only among five intermediate-risk pairs, with three reclassified as low-risk (i.e., an underestimation of risk) and two as high-risk (an overestimation of risk). Alloimmune risk categories were preserved in 90% of recipients (κ = 0.85, 95% CI 0.73–0.97) when all DRB4*01:01 alleles were imputed as DRB4*01:03. There was no significant difference in the misclassification rate between deceased-donor (n = 3/19, 16%), living-related (n = 1/24, 4%), and living-unrelated (n = 1/8, 13%) subgroups (p = 0.36).

**FIGURE 1:**
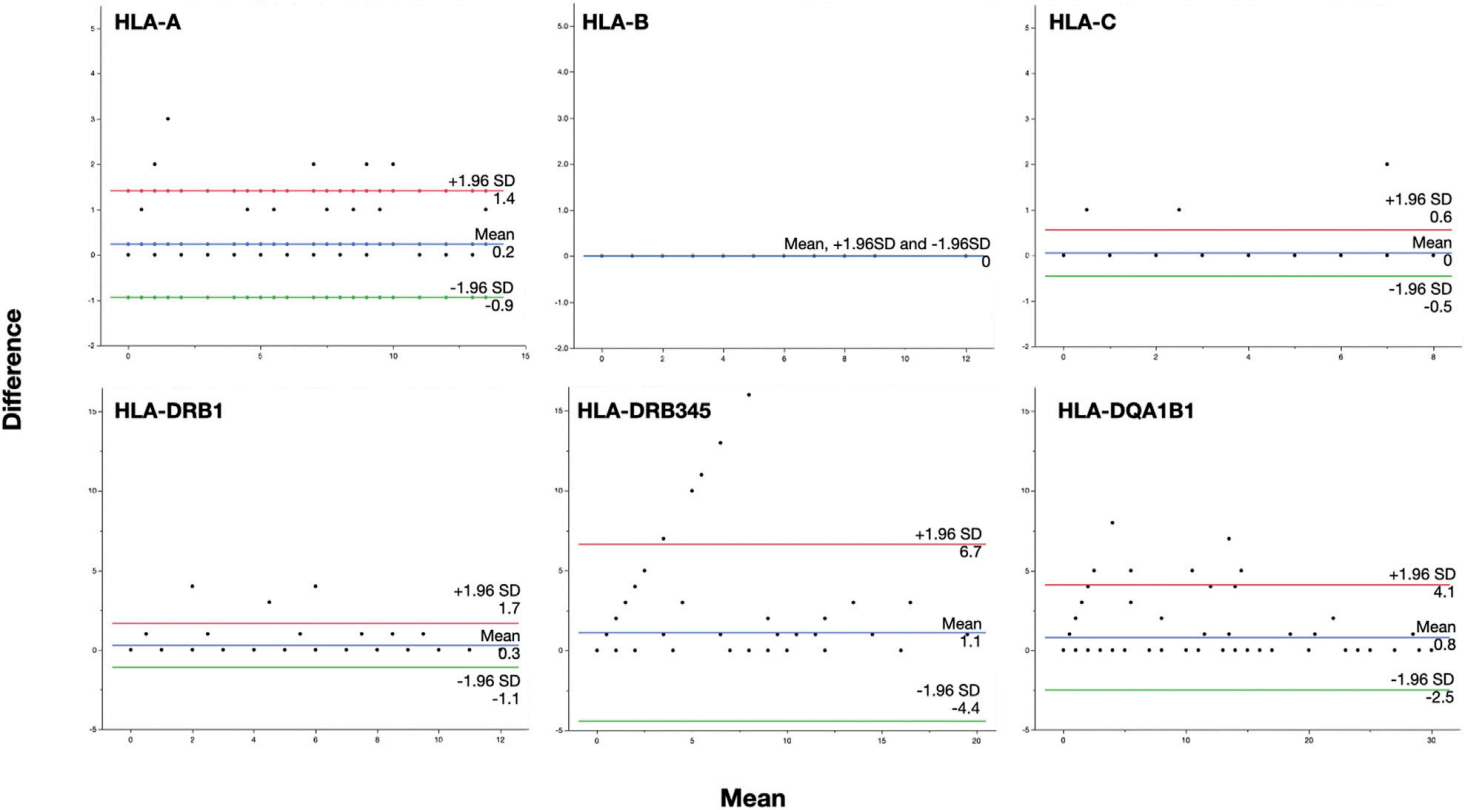
Bland-Altman plots of single-molecule eplet mismatches at each HLA locus. Bland-Altman plots showing agreement between donor-recipient single-molecule eplet mismatches calculated by NGS typing and imputation at each HLA locus. Each point represents the difference between methods plotted against their mean. The blue lines indicate the mean bias, and the red and green lines indicate the 95% limits of agreement (mean difference ±1.96SD). For the HLA-B locus, the mean bias and 95% limits of agreement are all zero. HLA, human leukocyte antigen; NGS, next-generation sequencing; SD, standard deviation.

Our findings are consistent with previous studies evaluating the impact of imputation on eplet mismatches. Cohen et al. reported a strong correlation for both class I and class II single-molecule eplet mismatches across races [6]. Senev et al. reported no difference in eplet mismatch load for 91.3% of imputed class I alleles, with 95.8% differing by at most 1 eplet. Although only 53.9% of imputed class II alleles had the same eplet mismatch load, 83.7% were within one eplet difference [5]. More importantly, the impact of imputation on alloimmune risk categorization was modest: 90% of recipients remained in the same category, and none were reclassified from low to high risk or *vice versa*. Although this cohort is underpowered to estimate the rate of clinically meaningful misclassification, these findings are consistent with those of Cohen et al., who reported that only 1/35 recipients changed from low- to intermediate risk, indicating that imputation preserved accurate mMM risk categorization.

In previous studies of imputation accuracy, cohorts were representative of European or North American populations. Few studies have been conducted in Southeast Asian cohorts that applied imputation to genotyped data using reference databases to derive Southeast Asian-centric reference panels [9, 10]. These reference-data limitations may account for the lower concordance for the imputation of Class II alleles. Our study, therefore, contributes to the existing literature by evaluating the performance of imputation tools in a multi-ethnic Southeast Asian cohort and by applying them to molecular mismatch.

Although next-generation sequencing remains the gold standard, our study provides exploratory data and proof of concept that imputation may be a reasonable and practical alternative for mMM risk classification in Southeast Asian populations, especially in historical cohorts and resource-limited settings where high-resolution data is unavailable or prohibitively costly. The use of transformed NGS-derived data, rather than genuinely low-resolution HLA typing, may limit the generalizability of the findings to routine clinical practice. Other limitations include the modest sample size, the absence of HLA-DP, given its emerging role in alloimmunity, and the lack of correlation with DSA and post-transplant outcomes. Future research should focus on developing region- and race-specific haplotype reference datasets to improve accuracy in multicultural populations. Nevertheless, imputation may represent a scalable approach to expanding access to mMM risk stratification.

## Data Availability

The raw data supporting the conclusions of this article will be made available by the authors, without undue reservation.
